# The feasibility of cell phone based electronic diaries for STI/HIV research

**DOI:** 10.1186/1471-2288-12-75

**Published:** 2012-06-12

**Authors:** Devon J Hensel, James D Fortenberry, Jaroslaw Harezlak, Dorothy Craig

**Affiliations:** 1 Section of Adolescent Medicine, Indiana University School of Medicine, Indianapolis, IN, USA; 2 Division of Biostatistics, Indiana University School of Medicine, Indianapolis, IN, USA; 3 Department of Sociology, Indiana University Purdue University-Indianapolis , Indianapolis, IN, USA

## Abstract

**Background:**

Self-reports of sensitive, socially stigmatized or illegal behavior are common in STI/HIV research, but can raise challenges in terms of data reliability and validity. The use of electronic data collection tools, including ecological momentary assessment (EMA), can increase the accuracy of this information by allowing a participant to self-administer a survey or diary entry, in their own environment, as close to the occurrence of the behavior as possible. In this paper, we evaluate the feasibility of using cell phone-based EMA as a tool for understanding sexual risk and STI among adult men and women.

**Methods:**

As part of a larger prospective clinical study on sexual risk behavior and incident STI in clinically recruited adult men and women, using study-provided cell phones, participants (N = 243) completed thrice–daily EMA diaries monitoring individual and partner-specific emotional attributes, non-sexual activities, non-coital or coital sexual behaviors, and contraceptive behaviors. Using these data, we assess feasibility in terms of participant compliance, behavior reactivity, general method acceptability and method efficacy for capturing behaviors.

**Results:**

Participants were highly compliant with diary entry protocol and schedule: over the entire 12 study weeks, participants submitted 89.7% (54,914/61,236) of the expected diary entries, with an average of 18.86 of the 21 expected diaries (85.7%) each week. Submission did not differ substantially across gender, race/ethnicity and baseline sexually transmitted infection status. A sufficient volume and range of sexual behaviors were captured, with reporting trends in different legal and illegal behaviors showing small variation over time. Participants found the methodology to be acceptable, enjoyed and felt comfortable participating in the study.

**Conclusion:**

Achieving the correct medium of data collection can drastically improve, or degrade, the timeliness and quality of an individual’s self-reported sexual risk behavior, which in turn, is a key factor in the success of intervention or education programs relying on this information. Our findings demonstrate that completion of electronic diaries via cellular phone is feasible way to describe STI/HIV risk among clinically recruited adult men and women.

## Background

Obtaining participant self-reports is common in STI/HIV research, not only because it is difficult to directly observe sexual risk behavior [1], but also because it is important to understand how an individual’s own circumstances and subjective experiences may increase or decrease the likelihood of a given behavior [2,3]. Eliciting accurate information is therefore to the vital to the correct design and implementation of intervention and education programs [3-5]; however, many of the self-reported sensitive, socially stigmatized or illegal behaviors collected in STI/HIV research can raise challenges in terms of the validity and reliability of data collected [1,6], particularly if behaviors are underreported out of participant concern for privacy or interviewer responses [6-8].

Recent studies suggest that the use of electronic data collection tools can increase the accuracy of self-disclosed risk behaviors by allowing a participant to self-administer a survey or diary, in their own environment, as close to the occurrence of the behavior as possible [9]. Ecological momentary assessment (EMA) [2,4,10-12] is one such tool, in which participants respond to pre-programmed signals on an electronic device (e.g., a PDA or cellular phone) prompting them to complete diaries, in various within-day frequencies, related to recent or immediate social environment and behavior [13,14]. This arrangement allows joint assessment of a risk behavior, and the context in which it occurs, in near real time [15]. Data collection typically proceeds over weeks or months [16,17], providing a better understanding of how social context differs when a behavior does and does not occur, as well as how the relationship between context and behavior may change over time.

EMA lends a number of advantages for use in clinical research. Compared to other collection modalities, it garners less missing data, higher reporting levels, stronger internal data validity and low behavior reactivity [4,8,12,14,15,18-28]. The pervasive use of cell phones and other electronic devices in the study population may make protocol training and data entry easier for participants [29,30]. Electronic platforms can time-stamp all data, from reminders to start/finish diaries, to actual initiation and completion, allows a precise analysis of participant compliance [4,8,14,18]. Moreover, researchers can standardize measures across participants and have increased flexibility, including using branching/contingency questions, or multilingual versions of instruments [4,12,18,19]. Finally, EMA strengthens the security of sensitive or stigmatizing information, allowing information to be vacated from the device immediately upon data entry for storage on a remote server, increasing participant valued privacy [20].

EMA has been utilized to examine a variety of clinical outcomes, in a number of high-risk populations, including cocaine relapse in homeless patients in treatment [17], adolescent mood variability and smoking [31], adolescent affect before and after sexual intercourse [15], prevention behavior in HIV-infected individuals [16], affect and drug craving in methamphetamine-dependent individuals [32] and women’s compliance to bacterial vaginosis (BV) treatment [33]. In this paper, we evaluate the use of cell-phone based, thrice-daily EMA over 12 weeks as a means of prospectively studying sexual risk behavior and incident STI among a sample of clinically recruited adult men and women at high risk for HIV/STI. Parallel to other work [34], we assess feasibility in terms of (a) compliance, or data entry which is consistent with study protocol and substantially complete; (b) reactivity, or behavior change attributed to study participation; (c) participant acceptability, or convenience and ease of method beneficial to compliance. We also assess (d) data capture, or the volume of sexual and risk behaviors collected. We also comment on participant recruitment and retention within a high-risk, clinically recruited population, as well as provide recommendations for future method application.

## Methods

### Study design

Data were collected as part of a larger 12-week study prospectively examining sexual risk behavior and incident STI in clinically recruited adult men and women. In this larger study, data collection was organized around three components: 1) an audio computer-assisted self-interview (ACASI),administered at enrollment and exit visits, assessing individual and partner-specific demographics, sexual and contraceptive history, general attitudes and relationship content for up to five partners; 2) thrice daily EMA diaries monitoring individual and partner-specific emotional attributes, non-sexual activities, non-coital or coital sexual behaviors, and contraceptive behaviors; 3) self-obtained urine (males) or vaginal specimens for STI diagnosis. The current study evaluates data taken primarily from the within-day diaries.

### Participants

Participants in the larger study were recruited from the patient population of the Bell Flower Clinic (BFC), a sexually transmitted diseases clinic operated by the Marion County Health Department in Indianapolis, Indiana. BFC serves primarily lower- and middle-income individuals residing in areas with high rates of unintended pregnancy and STI. Eligibility for the larger study included being 18 to 29 years of age and having primary residence in Marion County, Indiana for the 90 days of study duration. Both criteria were chosen to recruit a sample with a broad number of types of sexual relationships and reasons for sexual activity, as well as high rates of sexually transmitted infections, to facilitate follow up and to reduce sample attrition. Exclusion criteria included primary language other than English, ongoing participation in other research protocols at BFC, intoxication or psychiatric illness at enrollment, being HIV positive or being homeless.

At the time of analyses, all participants self-referred into the study based on study advertisements in BFC, as well or word of mouth within the patient population. All interested individuals contacted the research nurse practitioner (RNP), who provided additional study information, answered questions and performed initial screening. Very few (10%) of those calling were ineligible; of these, most exceeded the study age requirements, and one was HIV positive. Individuals who passed screening either scheduled an enrollment appointment at BFC within the next week, or were placed on a waiting list until a spot was available. The waiting list volume has ranged from 50 – 150 individuals, with a median time from placement on the waiting list until enrollment of four months. About 50% of individuals on the waiting list at any given time were never enrolled, most commonly due to an individual’s contact information changing between initial call and call-back. Very few were not enrolled due to a change in eligibility criterion. Retention and completion of enrolled participants is described in the results section.

### EMA diary technology

EMA diary data were entered on an internet enabled Palm Centro cellular phone, measuring 4.2 in × 2.1 in × 0.7 in. Each phone was equipped with a 1.3 megapixel digital camera, touch screen navigation, a QWERTY keyboard, as well as a carrying case, wall charger and a stylus to facilitate data entry. The diaries ran on software (Pendragon SyncServer) allowing Palm OS handhelds and smartphones to synchronize forms and data securely over a TCP/IP connection. Because the smartphones access a nationwide cellular network, internet connection is possible throughout the continental U.S. with access to cellular signals, considerably increasing efficiency in speed of data transfer. The software allowed users to connect and transfer information to the study database at any time, with multiple study participants able to connect simultaneously. The software enabled study personnel to amend data collection forms automatically, without any action from the user. Additionally, in the event of equipment or program failure, study personnel were able to reinstall the study application remotely.

Participants had an availability window to complete the diary from 30 minute prior to the scheduled start to four hours after the scheduled start. Prior to, or after this window, data collection was disabled. Participants also received up to four SMS text message reminders about completing diary entries, sent at the scheduled start time, one hour after the scheduled start time, two and a half hours after the scheduled start time and fifteen minutes before the 4-hour availability window following the scheduled start time closed. Reminders were sent until the data were entered *and* successfully downloaded. Thus, a participant who had taken their diary, but whose data did not successfully transfer off their phone would still receive a diary reminder, indicating they needed to troubleshoot connectivity with the RNP.

**Figure 1:**
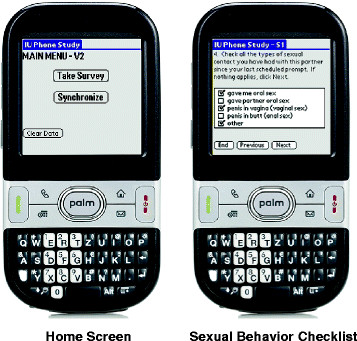
Example EMA diary screens: Home Screen for initiating survey and synchronizing data (left) and for selecting sexual behaviors (right).

Participants clicked to enter the survey on a Home Screen (Figure 1; left) and completed a specific sequence of questions assessing information since their last scheduled entry. This information included mood, and if any partner interaction had occurred. If they indicated no interaction, they were asked to identify, from a checklist, all the *individual* activities they had engaged in. If partner interaction did occur, they were asked to identify, from a checklist, any *partnered* sexual behaviors, that had occurred (e.g., Figure 1; right), as well as the number of times each behavior occurred. They also answered questions related to the order of *partnered* sexual behaviors, as well as use of contraception. Following these questions, participants were asked about any non-sexual *partnered* activities (e.g. shopping, playing a game, etc.). If no sexual activity was indicated, participants were directly taken to indicate which non-sexual partnered activities that occurred. Any sexual partners named in the enrollment ACASI were auto-populated into each participant’s cell phone for selection from a drop down menu, and participants could also free text the name of additional sexual partners.

Diaries were formatted to display one question on the screen at a time, requiring participants to select from a drop down list, or check appropriate boxes. Navigation from question to question was facilitated by the use of a “previous” and “next” button on each screen; however, for most questions, participants could not advance until the current question had been answered. For some sensitive questions, a “Prefer Not to Answer” option was available. These features were built into the diary program to reduce data errors and omissions.

In all data-related interactions, including actual data entry and reminders, time and date recorded and stored in the phone until transfer in real time to a remote server. Upon survey completion, participants were directed back to Home Screen (Figure 1; left) and clicked to synchronize data and initiate this transfer. Once transferred, participants could not enter the survey again until their next scheduled entry. In the event that connectivity issues prevented data transfer, information remained on the device, encrypted and password protected, until synchronization was next possible. In the event of equipment failure, the participant was instructed to contact the RNP, who verbally walked the participant through a procedure to clear the data from the phone and begin the data entry process over again. Information related to synchronization issues and data clearing was also time and date stamped for storage on the remote server.

### EMA diary protocol

At enrollment, following the informed consent process and ACASI, the RNP provided a face-to-face, in-depth orientation to basic cell phone operation, diary access and data entry, as well as policies on cellular service usage and troubleshooting procedures for equipment or connectivity issues. Participants were also given a detailed information sheet concerning all policies, as well as contact information for the RNP and research field staff. Additionally, weekly contact with research field staff during specimen collection enabled reinforcement of study procedures.

Participants completed EMA diaries three times per day, at eight hour intervals (e.g., 6am, 2pm and 10pm), over approximately 84 days, for a total of 252 entries per person. Although some EMA researchers suggest prompting diary completion at random intervals each day (e.g. [4]), recent work suggests that data collection at consistent times each day is equally valid [35], and may increase the convenience of, and compliance to, the data collection procedure for participants. Thus, participants selected one of four pre-set 8-hour diary entry schedules which fit best within their daily lives. They were also allowed to change their schedule at any time, however, very few (N = 4) opted to do so. All diary procedures, including data collection schedule and instrument comprehension were piloted with an initial sample (N = 10) of individuals from the target population for two weeks, with modification to a small number of items before total field deployment.

### Participant compensation

As part of the larger compensation structure for the study, participants received $1.00 per on–time diary entry, earning up to $252.00 for the diary portion during the entire study. Compensation was delivered via check in monthly installments, delivered by the research field staff. Additionally, entries which were delayed or missed due to connectivity issues or equipment failure were also compensated on a case-by-case basis. Participants also had access to unlimited domestic calling, text messages and phone-based internet use during the study. Those who successfully completed the study were given the option of retaining their study cell phone and paying for service themselves. Decisions to keep or not to keep the cell phone did not influence compensation from other parts of the study. The majority of fee-for-service options (e.g., games, directory assistance, international calling) was disabled from the phone; however, any charge incurred was deducted from the participant’s final paycheck.

### Diary measures

Partnered sexual behaviors (no/yes) included: received oral sex, gave oral sex, penile-vaginal sex, penile-anal sex and other sex. We also examined *individual* and *partnered* substance use (no/yes) including marijuana, alcohol or cocaine/methamphetamine use, as well as *individual* and *partnered* daily activities (no/yes) including taking a walk and going to the mall.

### Analyses

Descriptive statistics were used to examine compliance at the subject and partner level, completion time, acceptability and data capture. Reactivity was assessed using regression analyses adjusted for multiple diary entries within individuals [36].

## Results

### Overall participant retention

At the time of analyses, 277 participants had enrolled in the study, 87.7% (N = 243) completed all parts of the study and are used in the current paper. The non-completing group (N = 34; 12.2% of all enrolled) included four participants who failed to show for an exit interview, 11 who had participation suspended for short term incarceration (and finished participation after release). An additional 19 participants were withdrawn for extended incarceration (N = 5), for falling below 80% completion in the study and failing to respond to intervention efforts by the field staff (N = 8), or for failing to procure a police report within 72 hours of reporting a study phone as lost/stolen (N = 6). Rates of non-completion were commensurate with expected attrition within this population [37]. Descriptive analyses of the completing and non-competing groups suggest similar rates of gender (completers: 60.91% female; non-completers: 56.92% female), age (completers: 21.81% 20–22 years, 23.46% 22–24 years, 19.34% 24–26 years, 14.81% 26–28 years and 7.41% older than 28 years; non-completers: 20.92% 20–22 years, 22.15% 22–24 years, 18.77% 24–26 years, 15.69% 26–28 years and 7.69% older than 28 years) and race (completers: 87.65% African American; non-completers: 88.92% African American) differences. The characteristics of the sample are presented in Table 1.

**Table 1:** Demographic Characteristics and Infection Status, Completed Participants (N = 243)

**Variable**	**N (%)**
Gender	
Male	95 (39.09)
Female	148 (60.91)
Race	
White	19 (7.82)
African American/Black	213 (87.65)
Other	9 (3.70)
Don’t know	1 (0.41)
More than one race	1 (0.41)
Ethnicity	
Hispanic	8 (3.29)
Non-Hispanic	176 (72.02)
Not Available	60 (24.69)
Age	
Less than 20 year	32 (13.17)
20–22 years	53 (21.81)
22–24 years	57 (23.46)
24–26 years	47 (19.34)
26–28 years	36 (14.81)
28 or older	18 (7.41)
STI at Enrollment	
No	189 (77.78)
Yes	54 (22.22)
Chlamydia	26 (10.69)
Gonorrhea	7 (2.88)
Trichomoniasis	28 (11.52)
STI Acquisition during Study	
No	158 (65.02)
Yes	85 (34.97)
Gonorrhea	19 (7.81)
Chlamydia	37 (14.81)
Trichomoniasis	49 (20.16)

### Compliance

The complex structure of data collection required compliance analysis at three levels. First, participants were asked to complete three diary entries per day, resulting in 21 expected submissions *per week* during all 12 weeks of study participation. Second, each *participant* was expected to complete a total of 252 entries by the end of the study. Third, at the *study level*, the 243 completed participants were expected to contribute 61,236 (243 × 252) entries by the end of the study.

Compliance in the completing group was excellent. At the *weekly* level, participants submitted an overall average of 18.86 (SD = 2.69; Median = 20) of the 21 (85.7%) expected weekly diaries during all 12 weeks. At the *participant* level, the majority of the completing group (82.3%: 200/243) submitted nearly all (99%: 250/252) of the expected diaries at the end of the study. At the *study* level, *completing* participants submitted 89.7% (54,914/61,236) of the total diaries expected. We also evaluated study level compliance in the non-completing group, both in terms of the influence on total diary submission rates when included with the completing group, as well as when examining submission rates only among those who dropped out. At the study level, the completing and non-completing together (N = 277 total) submitted 85.4% (N = 59,967) of the 69,804 (277 × 252) diaries expected. Focusing solely on those who dropped out (N = 19), the average time from enrollment to dropout was 48.7 days (Median: 54.5 days; range: 12.0 to 84.0 days); at the study level, they submitted 69.6% (1424/2046) of the diaries expected at the point in the study at which they dropped.

Thus, based on the description, both demographic and completion rate, of the completer sample (N = 243) and the non-completer sample (N = 34), we concentrated the remaining analyses only on the completing group, as they provided full information at each study period in terms of answers to the survey questions. This full information did not require us to make assumptions about the nature of any missingness patterns (e.g., at random vs. non-ignorable), as it would with data not obtained from the non-completing group. This choice does not reduce the validity of our conclusions less valid; rather, it allows comparisons conditional on study completions.

Table 2 illustrates *weekly* compliance of the completing group in more detail. Within each week, compliance remains high, with average submissions between 18.51 entries (88.1% completion) and 19.51 entries (92.9% average completion) in the first through eleventh weeks, dropping slightly to 17.03 average diaries (81.1% average completion) submitted in the last week. In all study weeks, the median number of diaries submitted was 18 or higher, representing 90% completion. Focusing on age, gender, race/ethnicity and infection status influences on reporting (Table 3), small differences were noted between men and women, with men completing 0.5 fewer diaries per week. Participants reporting their race as ‘Other’ submitted fewer diaries than Whites or African Americans, and those between 22 and 24 years had the lowest weekly average as compared to other age groups. There was no difference between baseline STI status and weekly submission rates. Additionally, at the study level, participants indicated some type of partner interaction (sexual or non-sexual) on about 7.3% (4423/54,914) of all diary periods. At the subject level, participants reported an average of 40 such interactions (15%: 40/252), ranging from very little partner interaction (minimum: 1.00) to a large volume of interaction (128.5%: 324/252). The latter number suggests that some individuals many spend every diary period with at least one, if not more, partners.

**Table 2:** Weekly Number of Diaries Submitted, Completed Participants (N = 243)

	**No. of participants**	**Median**	**Mean (SD)**
Week 1	243	20	19.45 (2.06)
Week 2	243	20	19.51 (2.39)
Week 3	243	20	19.30 (2.13)
Week 4	243	20	19.20 (2.48)
Week 5	243	20	18.98 (2.62)
Week 6	243	20	19.03 (2.60)
Week 7	243	20	18.86 (2.66)
Week 8	243	19	18.73 (2.77)
Week 9	243	20	18.91 (2.31)
Week 10	242	20	18.81 (2.48)
Week 11	241	19	18.51 (2.85)
Week 12	242	18	17.03 (3.70)

**Table 3:** Diary submission patterns by completed participant (N = 243) gender, race, age and infection status

	**Total number**		**Weekly submitted diaries**	
	**Diaries**	**Weeks in Study**	**Median**	**Mean (SD)**
Gender				
Male	21,133	1139	19	18.55 (2.78)
Female	33,771	1772	20	19.03 (2.61)
Race				
White	4,244	228	20	18.61 (2.72)
African American/Black	48,294	2552	20	18.92 (2.66)
Other	2,366	131	19	18.06 (2.93)
Age				
Less than 20 years	7,289	384	20	18.98 (2.52)
20–22 years	11,926	632	20	18.87 (2.71)
22–24 years	12,674	684	20	18.53 (3.07)
24–26 years	10,638	563	20	18.90 (2.61)
26–28 years	8,224	432	20	19.04 (2.41)
28 year or older	4,153	216	20	19.23 (2.23)
Any STI at Enrollment				
No	42,019	2,231	20	18.83 (2.66)
Chlamydia	48,273	2,563	20	18.83 (2.71)
Gonorrhea	52,711	2,791	20	18.89 (2.66)
Trichomoniasis	47,868	2,543	20	18.81 (2.70)
Yes	12,193	644	20	18.93 (2.81)
Chlamydia	5,939	312	20	19.04 (2.58)
Gonorrhea	1,501	84	19	17.87 (3.59)
Trichomoniasis	6,344	332	20	19.11 (2.70)

### Completion time

We assessed completion time as the number of minutes elapsed from initiation of subject diary to synchronization with the server. For participants with submitted diaries, the median time to completion was 1.77 minutes, with slightly less time required for subjects who reported no partner interaction within a diary period (median: 1.68) and greater time required when partners were reported (median: 3.00). The median time from the start of the subject portion of the diary to the start of the partner portion of the diary was 0.68 minutes, with about 2.25 minutes required to complete and synchronize this latter part. We additionally conducted analyses to assess completion time as a function of sexual behavior, finding that reporting any type of sex added about a minute to overall diary completion time.

### Reactivity

**Figure 2:**
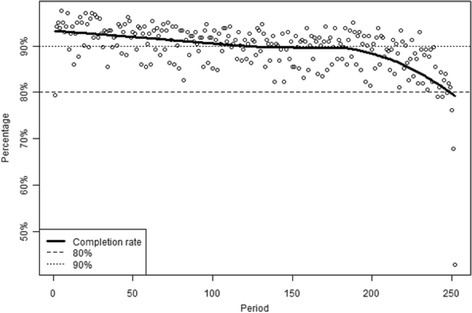
Trends in participant diary completion.

We examined two types of reactivity: completion reactivity and behavioral reactivity; that is, to what extent does diary completion and behavior reporting change simply as a function of real-time monitoring? First, as shown in Figure 2, within-diary completion submission rates ranged between about 80% and 93%, with general stability above 90% until over the halfway point of the study. This trend was significant, with a 0.61% decrease in submitted diaries with each passing week (*p <* .05).

**Figure 3:**
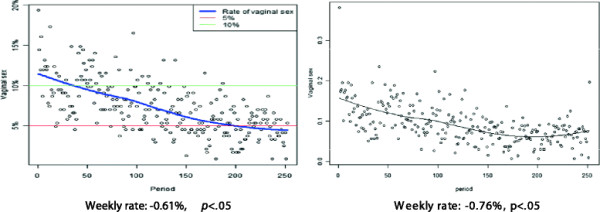
Trends in reports of vaginal sex, overall and by monogamous sexual relationships.

Next, we examined reports of vaginal sex, as well as two illegal “risk” behaviors, cocaine/methamphetamine use and marijuana use, one legal “risk” behavior, alcohol use, and one “neutral” behavior, taking a walk, over the 12 weeks of the study. As shown in Figure 3, overall reports of vaginal sex significantly declined by 0.61% each week (*p <* .05), resulting in about a 6% difference in reports between the first (11%) and last (5%) weeks in the study.

Similar significant declines (Figure 3) in vaginal sex were also noted within monogamous relationships. Controlling for gender, age and STI status, women experienced a significantly lower drop (rate: -0.50%) as compared to men (rate:-0.80%; *p <* .05) and those with no STI at enrollment (rate: -0.68%) decreased significantly more than those with an STI at enrollment (−0.36%; *p <* .05). Those of youngest age (18 to 20 years; rate: -0.91%) and oldest age (27 to 29 years; rate: -0.83%) reported significantly less frequently as compared to other age groups (21 to 23 years; rate: -0.41%; 24 to 26 years; rate: -0.49%; all *p* < .05).

**Figure 4:**
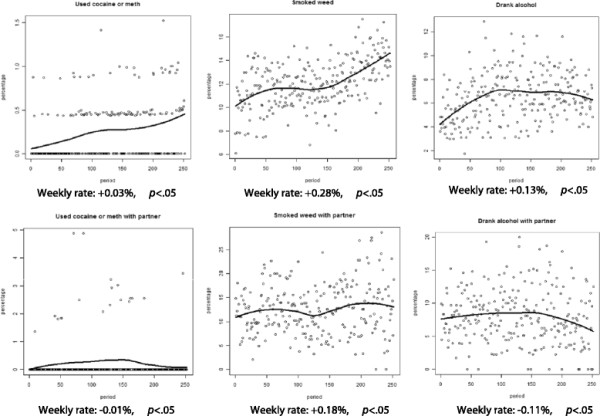
Trends in individual and partner-based risk behaviors: cocaine/methamphetamine use, marijuana use and alcohol use.

**Figure 5:**
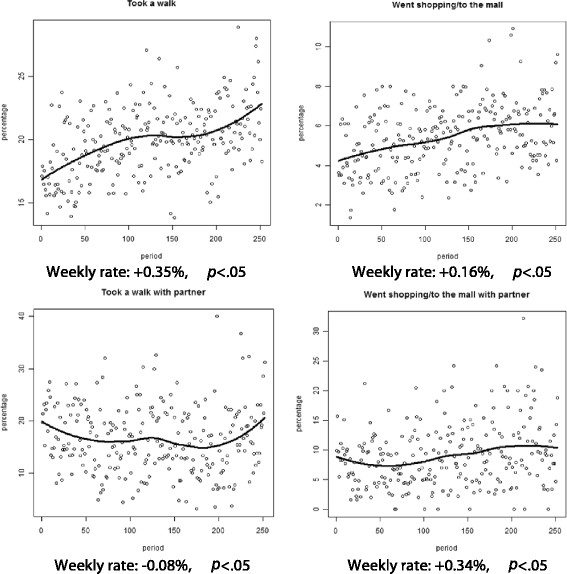
Trends in individual and partner-based neutral behaviors: taking a walk and going to the mall.

Additionally, as demonstrated in Figures 4 and 5, *individual/subject* reports of risk behaviors, including cocaine/methamphetamine use (0.03%), marijuana use (0.28%) and alcohol use (0.13%), as well as neutral behaviors, including taking a walk (0.35%) and going to the mall (0.16%), significantly increased per week in the same time frame. *Partner* based reports of cocaine/methamphetamine use (0.01%), alcohol use (0.11%) and taking a walk (0.08%) decreased, while marijuana use (0.18%) and going to the mall (0.34%) also significantly increased between the first and last weeks (all individual and partner effects *p <* .05).

### Acceptability

We assessed study acceptability using five, 5-point Likert items (strongly disagree to strongly agree) taken from the exit ACASI in which participants rated their study experience (Table 4). Participants highly rated the overall experience (Table 5) and a majority strongly agreed that they “enjoyed participating in the study.” Technical aspects of data collection were also highly rated: 63% strongly agreed that the “specimen collection procedure was convenient,” 78% strongly agreed that “questions were answered in a timely manner by the research team” and about 62% strongly disagreed that the “daily PDA questions were too long.” Almost 80% strongly agreed that they “did not change their behavior due to participating in the study.”

**Table 4:** Sexual event summary, by participants and events

	Total number participants reported*	Total number events reported**	**Median events per participants**
Received oral sex	229 (94.2)	1927 (43.5)	3.00
Gave oral sex	194 (79.8)	1454 (32.8)	2.00
Vaginal sex	243 (100.0)	3587 (81.1)	9.00
Anal sex	86 (35.3)	278 (6.2)	0.00
Other sex	151 (62.1)	1311 (29.6)	1.00

Percent of total sample completing the study; **Percent of total diaries with any partner interaction (N = 4423).

**Table 5:** Participant study experience evaluation

**Question**	**Mean (SD)**	**Median**
I enjoyed participating in the study*	4.74 (0.73)	5.00
I felt comfortable answering questions about sexual activities*	4.74 (0.67)	5.00
I did not change my behavior due to participating in the study*	4.56 (0.97)	5.00
The daily PDA questions were too long*	1.68 (1.10)	1.00
The specimen procedure was convenient*	4.27 (1.1))	5.00
Questions were answered in a timely manner by the research team*	4.64 (0.82)	5.00

*Ranged from 1 (Strongly Disagree) to 5 (Strongly Agree).

### Data capture

Commensurate with our presentation of the compliance data, we evaluated EMA’s capabilities as a data collection tool by examining the volume of sexual behavior data captured at the participant- and event-level (Table 4). At the participant level, all individuals reported vaginal sex some time during the 12 weeks. The majority also reported receiving oral sex (94.2%) or giving oral sex (79.8%). About 62% reported engaging in other types of sex, including about a third (35.3%) reporting any anal sex across the 12 weeks.

Similar patterns were observed in the event level data. Overall, participants submitted 4423 total entries noting with any partner interaction. Of these, the majority (81.1%: 3587/4423) were associated with vaginal sex. Oral genital sex, both receiving (43.5%: 1927/4423) and giving (32.8%: 1927/4423) were also common. Other sex represented about a third of all (1311/4423) of all interactions, while very few (6.2%) were associated with anal sex.

### Ease of use

Participants reported technical issues around connectivity and equipment loss/failure. Related to connectivity, the most common problems were inability to access the survey at some point (about 5% of participants) and difficulty synchronization of completed data with the remote server at some point (about 20% of participants). Upon experiencing connectivity issues, participants contacted either the RNP or other field staff, who verbally walked them through a reset procedure designed to save data until services were restored. In the rare event that the reset procedure did not work, the RNP contacted the information technology staff assigned to the study, who explored on site reasons for connectivity problems. Participants were compensated for missed surveys associated with connectivity.

Related to equipment loss/failure, the most common reasons for phone replacement were due to theft (about 10%) or damage to the phone (about 15%). About 11% of participants experienced random equipment failure not associated with negligence, theft or misuse. In all instances, participants were given a replacement phone and compensated for missed surveys, provided they contacted the RNP or field staff as soon as possible, and, in the case of theft, arranged to receive a police report on the stolen phone. If the participant was unable to verify theft with a police report, they were removed from the study and not compensated for the missed surveys. The average time from report of issue to replacement phone was two days, with the most common resolution of field staff’s bringing the phone to the participant. Despite these technical issues, no participants left the study specifically due to issues using the cell phone and only 6 participants had to be removed because they could not produce a police report. All other technical problems were described by participants as minor issues associated with study participation.

## Discussion

Achieving the correct medium of data collection can drastically improve, or degrade, the timeliness and quality of an individual’s self-reported sexual risk behavior [20,38-40], which in turn can play a key factor in the success of intervention or education programs relying on this information. This is one of the first studies to evaluate the use of EMA to prospectively capture risk behavior in a high-risk, clinically recruited sample of adult men and women. Contrary to assumptions about challenges associated with recruitment and retention of this population in high-involvement studies [16,41], our findings demonstrate that completion of thrice daily, electronic diaries via cellular phone is feasible way to describe STI/HIV risk in clinical and/or high risk population, capturing an ample participant- and event-level volume of different sexual behaviors. Participants were highly compliant with diary entry protocol and schedule, found the methodology to be acceptable, enjoyed and felt comfortable participating in the study, and did not alter their risk behaviors as a result of frequent reporting. In addition, study provided phones and cellular service provided an added participation incentive and facilitated communication between participants and study staff.

Behavior reactivity can be a concern in EMA studies, particularly because behavior assessments are completed repeatedly and in proximity to the behavior of interest, putting them in a prime position to affect behavior [11]. Sexual behavior, drug or alcohol use and other “undesirable” behavior may be particularly vulnerable to reactivity [42]. However, most EMA studies show minimal to non-existent reactivity, even for sensitive and stigmatized behaviors [16,21,34,43]. Our data do suggest small reporting differences over time in a variety of in a variety of partnered and unpartnered, as well as legal and illegal behaviors, with most exhibiting less than one-half percent change per week. The most marked trend was a significant decrease in reports of vaginal sex, with these trends holding consistent across relationship types, age, gender and STI status. While no studies have assessed reactivity to sexual behaviors using EMA, our data are in line with reported reductions in longer EMA studies of alcohol use [44] and drug use [11]. Some work suggests that the same EMA data collection intensity that causes concern may in fact attenuate reactivity by causing habituation [45]; however, it is likely that these findings reflect subtle shifts in day-to-day routines, given both the high level of diary submission as well as the majority of those who reported no alternation of behavior as a result of study participation.

With EMA, a maximum of 20% missed or missing entries, or, 80% compliance, is considered satisfactory [46], as the chance of systematic bias in the data increases with higher noncompliance [34]. Our weekly (85.7%), subject (82.3%) and study (89.7%) diary completion levels are on par with or are higher than other shorter-term EMA studies with similar [32,47,48] or more frequent [17,49] reporting schedules, as well as EMA studies with once or twice per day reporting [16,34,50] and retrospective [20] or end-of-day [33] electronic diaries. Moreover, even with a larger number of items to complete as compared to other studies(e.g., [47], participants did not feel the diaries were too long, and they completed diaries at similar levels regardless of whether any partner interaction occurred and/or if any sexual behavior was reported.

It is well established in multiple samples that participant acceptability is generally not a problem [8]. The same can be said about our clinically recruited, high-STI risk sample of adult men and women, as the majority of individuals did not find the intense diary collection schedule to be burdensome. However, we argue that it is important to understand *method* acceptability in light of *other* aspects of study acceptability. Existing work emphasizes that participants value regular communication, health and social support from research/field staff, as well as compensation and study convenience, as important pieces in the quality of their study participation and their long term retention [41,51-57]. From this perspective, we believe that neither our sample’s high rating of within-day diary collection, nor their high compliance, is surprising, as they also rated their overall study experience, their connection with field staff and the convenience of data collection as high.

## Conclusion

The ability to keep participants engaged in HIV/STI research over an extended period of time is vital to capturing not only a sufficient volume of behavioral and epidemiologic data, but also to collect data that accurately reflect the habitude of the participants themselves. In contrast to assumptions about the difficulty of electronic and/or technically demanding data collection in clinical and high-risk samples [16,41], we suggest that, with the correct support structure, EMA is a feasible, highly acceptable, and effective tool for prospectively capturing sexual risk behavior and STI in these populations.

### Limitations

Comment is warranted on the representativeness of this sample. Recruitment did not occur exclusively from the active patient population at the county health clinic (BFC). Individuals, many of whom had sought services from BFC in the past, self-referred for enrollment in the study. All of these individuals resided in low-income areas associated with high levels of unintended pregnancy and STI. Based on the sample characteristics in prior studies conducted at BFC, as well as within the general BFC patient population, our participants do not substantially differ in terms of education, sexual behaviors, early child bearing or STI. Moreover, between 5% and 20% of participants did experience a variety of expected technical and equipment issues, mainly related to loss of connectivity, inability to synchronize data, random equipment failure or equipment loss/theft. While these issues all impact a participant’s ability to complete diary entries, and may influence their desire for continued study participation, we were able to minimize the impact of these issues by not only providing them prompt resources to address the concern, but also compensating them for entries missed due to connectivity. The majority of those who experienced issues were resolved within the same day. Additionally, our attrition rate is similar to, or lower than, other short term follow up studies in this clinic [37]. From this perspective, our exclusion criteria notwithstanding, we believe our data provide information that is generalizable to other clinic-based samples, particularly as a means of providing additional information on the processes of sexuality, sexual behavior and STI in higher risk persons (those economically disadvantaged, racial/ethnic minority, or both) whose risk is epidemiologically emphasized, but whose sexual relationships are largely ignored.

## Competing interests

The authors declare that they have no competing interests.

## Authors’ contributions

All authors participated in the conception and design of the study. JH conducted data analysis. DJH and JDF drafted the manuscript, and all authors critically revised and approved the final manuscript.

## Pre-publication history

The pre-publication history for this paper can be accessed here:

http://www.biomedcentral.com/1471-2288/12/75/prepub

## References

[B1] Catania JA Gibson DR DD C Methodological problems in AIDS behaviour research: influences on measurement error and participation bias in studies of sex behaviour Psychological Bulletin 1990 108 339 362 2270232 10.1037/0033-2909.108.3.339

[B2] Shiffman S Stone AA Introduction to the special section: Ecological momentary assessment in health psychology Health Psychology 1998 17 1 3 5

[B3] Scott-Sheldon LAJ Kalichman SC Carey M Freeland KE, Jennings JR, Llabre MM, Manuck SB, Susman EJ Assessment of Sexual behavior Handbook of Behavioral Medicine: Methods and Applications 2010 Springer, London 59 72

[B4] Shiffman S Stone A Hufford M Ecolocial Momentary Assessment Annual Review of Clinical Psychology 2008 4 1 32 10.1146/annurev.clinpsy.3.022806.091415 18509902

[B5] van Griensven F Naorat S Kilmarx PH Jeeyapant S Manopaiboon C Chaikummao S Jenkins RA Uthaivoravit W Wasinrapee P Mock PA Palmtop-assisted Self-Interviewing for the Collection of Sensitive Behavioral Data: Randomized Trial with Drug Use Urine Testing American Journal of Epidemiology 2006 163 3 271 278 16357109 10.1093/aje/kwj038

[B6] Catania JA Binson D VdS A Methodological research on sexual behavior in the AIDS era Annual Review of Sex Research 1995 16 77 125

[B7] Fenton KA Johnson AM McManus S Erens B Measuring sexual behaviour: methodological challenges in survey research Sexually Transmitted Infections 2001 77 2 84 10.1136/sti.77.2.84 11287683 PMC1744273

[B8] Bolger N Davis A Rafaeli E Diary Methods: Capturing Life as it is Lived Annual Review of Psychology 2003 54 579 616 10.1146/annurev.psych.54.101601.145030 12499517

[B9] Phillips AE Gomez GB Boily M-C Garnett GP A systematic review and meta-analysis of quantitative interviewing tools to investigate self-reported HIV and STI associated behaviours in low- and middle-income countries International Journal of Epidemiology 2010 39 6 1541 1555 10.1093/ije/dyq114 20630991

[B10] Stone A Shiffman S Ecological momentary assessment (EMA) in behavorial medicine Annual of Behavioral Medicine 1994 16 3 199 202

[B11] Shiffman S Ecological momentary assessment (EMA) in studies of substance use Psychological Assessment 2009 21 4 486 497 19947783 10.1037/a0017074 PMC2846437

[B12] Shiffman S Real-time self-report of momentary states in the natural environment: Computerized ecological momentary assessment 2000 Lawrence Earlbaum Associates, Mahwah, NJ

[B13] Piasecki TM Hufford MR Solhan M Trull TJ Assessing clients in their natural environments with electronic diaries: Rationale, benefits, limitations, and barriers Psychological Assessment 2007 19 1 25 43 17371121 10.1037/1040-3590.19.1.25

[B14] Hufford M Stone AA, Shiffman S, Atienza A, Nebeling L Special methodological challenges and opportunities in ecological momentary assessment The science of real-time data capture: Self-reports in health research edn 2007 Oxford University Press, Oxford, England 54 75

[B15] Shrier L Koren S Aneja P de Moor C Affect Regulation, Social Context, and Sexual Intercourse in Adolescents Archives of Sexual Behavior 2010 39 3 695 705 10.1007/s10508-008-9394-1 18818996

[B16] Cook PF McElwain CJ Bradley-Springer LA Feasibility of a daily electronic survey to study prevention behavior with HIV-infected individuals Research in Nursing & Health 2010 33 3 221 234 10.1002/nur.20381 20499392

[B17] Freedman MJ Lester KM McNamara C Milby JB Schumacher JE Cell phones for ecological momentary assessment with cocaine-addicted homeless patients in treatment Journal of Substance Abuse Treatment 2006 30 2 105 111 10.1016/j.jsat.2005.10.005 16490673

[B18] Richens J Copas A Sadiq ST Kingori P McCarthy O Jones V Hay P Miles K Gilson R Imrie J A randomised controlled trial of computer-assisted interviewing in sexual health clinics Sexually Transmitted Infections 2010 86 4 310 314 10.1136/sti.2010.043422 20551234

[B19] Trull TJ Ebner-Priemer UW Using experience sampling methods/ecological momentary assessment (ESM/EMA) in clinical assessment and clinical research: Introduction to the special section Psychological Assessment 2009 21 4 457 462 19947780 10.1037/a0017653 PMC4255457

[B20] Lim MSC Sacks-Davis R Aitken CK Hocking JS Hellard ME Randomised controlled trial of paper, online and SMS diaries for collecting sexual behaviour information from young people Journal of Epidemiology and Community Health 2010 64 10 885 889 10.1136/jech.2008.085316 19767322

[B21] Hufford MR Shields AL Shiffman S Paty J Balabanis M Reactivity to ecological momentary assessment: An example using undergraduate problem drinkers Psychology of Addictive Behaviors 2002 16 3 205 211 12236455

[B22] Blondin J Abu-Hasaballah K Tennen H Lalla R Electronic versus paper diaries: a pilot study of concordance and adherence in head and neck cancer patients receiving radiation therapy Head & Neck Oncology 2010 2 1 29 10.1186/1758-3284-2-29 20955592 PMC2972290

[B23] Luckmann R Vidal A Design of a handheld electronic pain, treatment and activity diary Journal of Biomedical Informatics 2010 43 5, Supplement 1 S32 S36 10.1016/j.jbi.2010.05.005 20937483

[B24] Epstein DH Willner-Reid J Vahabzadeh M Mezghanni M Lin J-L Preston KL Real-Time Electronic Diary Reports of Cue Exposure and Mood in the Hours Before Cocaine and Heroin Craving and Use Arch Gen Psychiatry 2009 66 1 88 94 10.1001/archgenpsychiatry.2008.509 19124692 PMC2943840

[B25] Tideman RL Pitts MK Fairley CK Client acceptability of the use of computers in a sexual health clinic Int J STD AIDS 2006 17 2 121 123 10.1258/095646206775455793 16464275

[B26] Turner CF Rogers SM Hendershot TP Miller HG Thornberry JP Improving representation of linguistic minorities in health surveys Public Health Reports 1996 111 3 276 279 8643822 PMC1381773

[B27] Ghanem KG Hutton HE Zenilman JM Zimba R Erbelding EJ Audio computer assisted self interview and face to face interview modes in assessing response bias among STD clinic patients Sexually Transmitted Infections 2005 81 5 421 425 10.1136/sti.2004.013193 16199744 PMC1745029

[B28] Langhaug LF Sherr L Cowan FM How to improve the validity of sexual behaviour reporting: systematic review of questionnaire delivery modes in developing countries Tropical Medicine & International Health 2010 15 3 362 381 10.1111/j.1365-3156.2009.02464.x 20409291 PMC3321435

[B29] Collins RL Kashdan TB Gollnisch G The feasibility of using cellular phones to collect ecological momentary assessment data: Application to alcohol consumption Experimental and Clinical Psychopharmacology 2003 11 1 73 78 12622345 10.1037//1064-1297.11.1.73

[B30] Fairley CK Sze JK Vodstrcil LA Chen MY Computer-Assisted Self Interviewing in Sexual Health Clinics Sexually Transmitted Diseases 2010 37 11 665 668 10.1097/OLQ.0b013e3181f7d505 20975481

[B31] Weinstein SM Mermelstein R Shiffman S Flay B Mood variability and cigarette smoking escalation among adolescents Psychology of Addictive Behaviors 2008 22 4 504 513 19071975 10.1037/0893-164X.22.4.504 PMC2605639

[B32] Galloway GP Didier R Garrison K Mendelson J Feasibility of Ecological Momentary Assessment Using Cellular Telephones in Methamphetamine Dependent Subjects Substance Abuse: Research and Treatment 2008 1 9 14 19997532 10.4137/sart.s428 PMC2789561

[B33] Bartley J Ferris D Allmond L Personal digitals assistants used to document compliance of bacterial vaginosis treatment Sexually Transmitted Diseases 2004 31 488 491 10.1097/01.olq.0000135990.21755.51 15273582

[B34] Sonnenschein M Sorbi MJ van Doornen LJP Maas CJM Feasibility of an Electronic Diary in Clinical Burnout International Journal of Behavioral Medicine 2006 13 4 315 319 10.1207/s15327558ijbm1304_6 17228989

[B35] Tennen H Affleck G Coyne JC Larsen RJ DeLongis A Paper and plastic in daily diary research: Comment on Green, Rafaeli, Bolger, Shrout, and Reis (2006) Psychological Methods 2006 11 1 112 118 16594771 10.1037/1082-989X.11.1.112

[B36] R Development Team R: A language and environment for statistical computing 2010 R Foundation for Statistical Computing, Vienna, Austria

[B37] Schillinger JAMDM Kissinger PP Calvet HM Whittington WLH Ransom RLM Sternberg MRP Berman SMMDS Kent CKM Martin DHM Oh MKM Patient-Delivered Partner Treatment With Azithromycin to Prevent Repeated Chlamydia trachomatis Infection Among Women: A Randomized, Controlled Trial Sexually Transmitted Diseases 2003 30 1 49 56 10.1097/00007435-200301000-00011 12514443

[B38] Minnis AM Padian NS Reliability of adolescents’ self-reported sexual behavior: a comparison of two diary methodologies Journal of Adolescent Health 2001 28 5 394 403 10.1016/S1054-139X(00)00218-4 11336869

[B39] Morrison DM Leigh BC Rogers Gillmore M Daily Data Collection: A Comparison of Three Methods Journal of Sex Research 1999 36 1 76 81 10.1080/00224499909551970

[B40] Shrier LA Shih M Beardslee WR Affect and sexual behavior in adolescents: a review of the literature and comparison of momentary sampling with diary and retrospective self-report methods of measurement Pediatrics 2005 115 5 e573 e581 10.1542/peds.2004-2073 15867022 PMC1570185

[B41] Yancey AK Ortega AN Kumanyika SK Effective recruitment and retention of minority research participants Annual Review of Public Health 2006 27 1 1 28 10.1146/annurev.publhealth.27.021405.102113 16533107

[B42] McFall RM Stuart RB Parameters of self-monitoring Behavioral self-management: Strategies, techniques, and outcome 1977 Brunner/Mazel, New York 196 214

[B43] Stone AA Broderick JE Schwartz JE Shiffman S Litcher-Kelly L Calvanese P Intensive momentary reporting of pain with an electronic diary: reactivity, compliance, and patient satisfaction Pain 2003 104 1–2 343 351 12855344 10.1016/s0304-3959(03)00040-x

[B44] Collins RL Morsheimer ET Shiffman S Paty JA Gnys M Papandonatos GD Ecological momentary assessment in a behavioral drinking moderation training program Experimental and Clinical Psychopharmacology 1998 6 3 306 315 9725114 10.1037//1064-1297.6.3.306

[B45] Moos RH Commentary. Context and mechanisms of reactivity to assessment and treatment Addiction 2008 103 2 249 250 18199303 10.1111/j.1360-0443.2007.02123.x

[B46] Stone AA Shiffman S Capturing Momentary, Self-Report Data: A Proposal for Reporting Guidelines Annals of Behavioral Medicine 2002 24 3 236 10.1207/S15324796ABM2403_09 12173681

[B47] Stone AA Shiffman S Schwartz JE Broderick JE Hufford MR Patient compliance with paper and electronic diaries Controlled Clinical Trials 2003 24 2 182 199 10.1016/S0197-2456(02)00320-3 12689739

[B48] Hong J Jiangna H Zhu Z Wenbin X Jinping Z Yuanjue Z Patient Compliance with Assessing and Monitoring of Asthma Journal of Asthma 2009 46 10 1027 1031 10.3109/02770900903229685 19995141

[B49] Litcher-Kelly L Kellerman Q Hanauer SB Stone AA Feasibility and Utility of an Electronic Diary to Assess Self-Report Symptoms in Patients With Inflammatory Bowel Disease Annals of Behavioral Medicine 2007 33 2 207 212 10.1007/BF02879902 17447873

[B50] Palermo TM Valenzuela D Stork PP A randomized trial of electronic versus paper pain diaries in children: impact on compliance, accuracy, and acceptability Pain 2004 107 3 213 219 10.1016/j.pain.2003.10.005 14736583

[B51] Gauthier MA WP C Gaining and sustaining minority participation in longitudinal research projects Alzheimer Dis Assoc Disord 1999 13 S29 S33 10369515 10.1097/00002093-199904001-00008

[B52] Hessol NA Schneider M Greenblatt RM Bacon M Barranday Y Holman S Robison E Williams C Cohen M Weber K Retention of Women Enrolled in a Prospective Study of Human Immunodeficiency Virus Infection: Impact of Race, Unstable Housing, and Use of Human Immunodeficiency Virus Therapy Am J Epidemiol 2001 154 6 563 573 10.1093/aje/154.6.563 11549562

[B53] Kimlin A-G Patricia AG Effect of time incentives on subject participation in a study of long-term breast cancer survivors: Are there ethnic differences? Journal of the National Medical Association 2000 92 11 528 11152085 PMC2568330

[B54] Parra-Medina D D’Antonio A Smith SM Levin S Kirkner G Mayer-Davis E Successful recruitment and retention strategies for a randomized weight management trial for people with diabetes living in rural, medically underserved counties of South Carolina: the POWER study Journal of the American Dietetic Association 2004 104 1 70 75 10.1016/j.jada.2003.10.014 14702587

[B55] Zayas LH McKee MD Jankowski KRB Adapting Psychosocial Intervention Research to Urban Primary Care Environments: A Case Example Annals of Family Medicine 2004 2 5 504 508 10.1370/afm.108 15506589 PMC1466732

[B56] Connell CM Shaw BA Holmes SB Foster NL Caregivers‘attitudes toward their family members’ participation in Alzheimer disease research: implications for recruitment and retention Alzheimer Dis Assoc Disord 2001 15 137 145 10.1097/00002093-200107000-00005 11522931

[B57] Russell C Palmer JR Adams-Campbell LL Rosenberg L Follow-up of a Large Cohort of Black Women Am J Epidemiol 2001 154 9 845 853 10.1093/aje/154.9.845 11682367

